# Preeclampsia and long-term risk of cardiovascular disease: what do obstetrician-gynecologists know?

**DOI:** 10.1186/1471-2393-13-61

**Published:** 2013-03-09

**Authors:** May-Britt Heidrich, Daniela Wenzel, Constantin S von Kaisenberg, Cordula Schippert, Frauke M von Versen-Höynck

**Affiliations:** 1 Department of Obstetrics and Gynecology, Hannover Medical School, Carl-Neuberg-Str. 1, Hannover, 30625, Germany; 2 Department of Biostatistics, Hannover Medical School, Carl-Neuberg-Str. 1, Hannover, 30625, Germany

**Keywords:** Preeclampsia, Follow up care management, Cardiovascular risk, Guidelines

## Abstract

**Background:**

Preeclampsia (PE), a hypertensive disorder of pregnancy affects 2-8% of women and is associated with increased cardiovascular disease (CVD) risk later in life. There is little information about the knowledge of obstetrician-gynecologists in German outpatient care setting regarding the future health risk of PE and knowledge of the current guidelines on treatment and counseling patients post PE. This study aimed to assess whether obstetrician-gynecologists are aware of PE’s association with maternal long-term adverse outcomes and providing appropriate counseling.

**Methods:**

A random sample of 500 obstetrician-gynecologists in the federal state of Lower Saxony was mailed a survey and a reminder with a second copy of the survey. The questionnaire elicited both personal information, and knowledge on future disease risks, e.g. cardiovascular disease (CVD) and current guidelines as well as on counseling practice. Descriptive analysis was used to analyze the responses.

**Results:**

A total of 212 obstetrician-gynecologists (42.4%) responded to the questionnaire. A large proportion of physicians stated that PE was associated with a higher risk for the development for hypertension (86.6%), stroke (78.5%) and kidney disease (78.0%). Of the participants 75.8% reported that women after PE have a shorter life expectancy. Respondents with knowledge of the current guidelines of the German Association of Obstetrics and Gynecology concerning follow up and risk management of PE (45.2%) were more often aware of the development of CVD and stroke and counseled patients on self -blood-pressure measurement, meaning and long-term-risks of PE and attached importance to family history of PE compared to physicians with no knowledge of the guidelines.

**Conclusion:**

Although the majority of obstetrician-gynecologists were aware of higher CVD risk after PE, weaknesses exist in the follow up care and counseling of these patients. These deficiencies would be amendable to directed educational activities to improve the implementation of current guidelines.

## Background

Preeclampsia (PE) is a hypertensive disorder of pregnancy that effects 2-8% of pregnant women and typically presents with proteinuria and hypertension in the second half of pregnancy [1,2]. PE is one of the leading causes of maternal and neonatal morbidity and mortality [3].

PE is associated with abruption or infarction of the placenta, preterm birth and can be further complicated by fetal growth restriction and intrauterine fetal death [4]. Most of the pathological conditions associated with PE appear to resolve after delivery. However there is growing evidence that women with a history of PE are more likely to develop cardiovascular disease (CVD) later in life [5]. Long-term studies suggest an increased risk of stroke [6-9], ischemic heart disease [10] and type II diabetes [11] later in life among women with a history of PE. A history of PE marks an increased risk of future chronic kidney disease [12] and shorter life expectancy [13-15].

 CVD is a leading cause of mortality among women in Germany [16]. Large cohort studies indicate that a woman’s obstetrical history is an important part of her risk profile for future CVD, and PE as a risk factor for CVD with an up to eightfold increased risk compared to women with no history of PE [17,18].

The national guideline for the primary prevention of CVD does not address the association between PE and future CVD in any form [19]. The German Association of Gynecology and Obstetrics (DGGG) refers in its guidelines to an increased risk of the later development of CVD for women who experienced PE and recommends to evaluate renal function 3 months post partum, blood pressure monitoring post partum until normalization of the blood pressure and the woman’s detailed information about her higher CVD risk [20].

 We hypothesized that gynecologists and obstetricians in the outpatient setting have limited knowledge about the association of PE with future CVD and the current recommendation for the follow up and counseling of women that experienced PE. Thus, we sought to determine to what extend gynecologists in the public state of Lower Saxony (Germany) are aware of this association and whether they were providing appropriate counseling and care to women after experiencing PE.

## Methods

An anonymous survey and a second reminder with a copy of the questionnaire were sent to a random sample of 500 out of 885 gynecologists and obstetricians working in an outpatient setting in Lower Saxony. The physicians were randomly selected to participate in the study on basis of an address directory existing for all obstetricians/gynecologists in Lower Saxony.

The survey responses were collected between January and March 2012. The survey contained a total of 16 questions to elicit information about the personal characteristics of the obstetrician-gynecologist, their knowledge, especially with regard to the relationship between PE and future disease risks, e.g. hypertension, stroke and renal diseases, as these outcomes were studied by other researchers and cohort studies.

The survey also contained questions on the providers typical counseling for cardiovascular risk reduction among women with a history of PE. Finally it included two long-term outcomes -malignancy and liver disease - that are not associated with PE. The translated survey distributed to the practitioners is given in Additional file 1. The local Ethical Committee of Hannover Medical School approved the study.

### Statistical analyses

Data are presented descriptively, providing mean values and standard deviations or percentages. The differences between the case groups were investigated with Chi-Square or Fisher exact test. In case of low numbers in the separate groups answers were grouped according to frequency of occurrence of positive/negative responses. The alpha level was set at 0.05. Data were analyzed using SPSS for Windows, Version 18.0 (SPSS Inc., Chicago, USA).

## Results

A total of 212 gynecologists completed the survey, yielding a response rate of 42.4%. Out of 261 female physicians 135 (65.92%) and of 239 male physicians 70 (29.28%) responded. The majority of physicians had 11-20 years of clinical experience (Table 1).

**Table 1:** Demographic and outpatient practice characteristics

**Characteristics of respondents**	
**Years of clinical experience**	
≤ 10 years	n=36 (17.5%)
11-20 years	n=97 (46.6%)
≥ 21 years	n=75 (36.1%)
**Sex**	
Male	n=70 (34.1%)
Female	n=135 (65.9%)
**Proportion of treated patients** >**50 years**	
≤ 10%	n=17 (8.3%)
11-30%	n=102 (49.8%)
31-50%	n=72 (35.1%)
≥ 51%	n=14 (6.8%)

### Medical history and counseling of women with a history of PE

**Figure 1:**
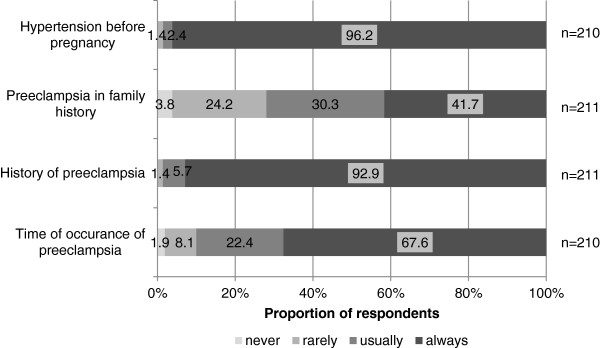
Analysis of physician’s responses concerning the medical history they take from their patients.

**Figure 2:**
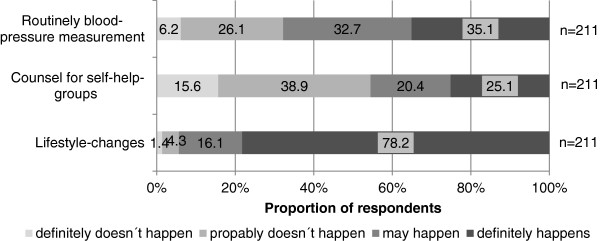
Analysis of physician’s counseling behavior.

Nearly all gynecologists included preexisting hypertension (98.6%) and PE (98.6%) as part of their medical-history, including family-history of PE (72.0%) and the time of PE-development during pregnancy (90.0%), (Figure 1). The majority (71.3%) of physicians counseled women >50 years about CVD risk. Of all respondents 65.3% provided counseling to patients with a history of PE about the increased risk of CVD. The regular measurement of blood pressure was recommended by 67.8% of obstetrician-gynecologists to women after PE. Of all participants 94.3% included recommendations for life-style changes in their counseling strategy (Figure 2).

### Follow up care and risk factor management after PE

**Figure 3:**
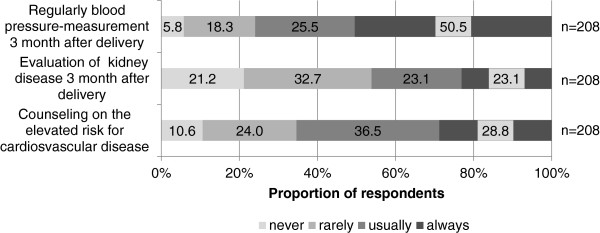
**Analysis of follow**-**up care in the outpatient setting after preeclampsia.**

Blood pressure measurement three months post partum as recommended by the current German guidelines was performed by 76.0% of all gynecologists that work in an outpatient setting. The development of kidney disease was evaluated by 46.2%, (Figure 3).

### Knowledge of the association of PE and future vascular disease

**Figure 4:**
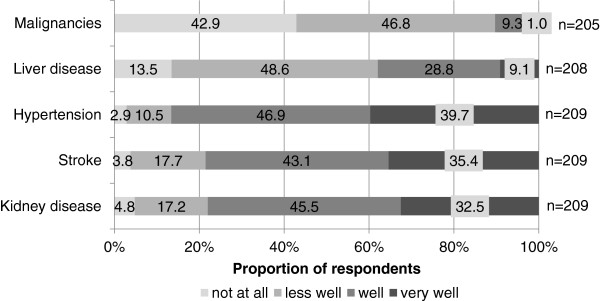
Physicians knowledge of future health risks after a history of preeclampsia.

A high percentage of gynecologists that work in an outpatient setting had knowledge about a higher risk for the development of hypertension (86.6%), gave respect to the risk of the development of stroke (78.5%) and were aware of an increased risk of kidney disease (78.0%) after PE. Of all respondents 75.8% were correct about the association of PE and shorter life expectancy (Figure 4).

### Knowledge of The Guidelines of the German Association of Gynecology and Obstetrics (DGGG)

Less than half (45.2%) of the respondents were aware of the current guidelines concerning the follow up and risk management of PE. To evaluate whether there is a causal relationship of the physicians knowledge of national guidelines and their follow up treatment we created two subgroups. Physicians that indicated that they were very well or well familiar with the current guidelines were grouped together (physicians with knowledge, N=94) and physicians that stated that they were less well or not at all familiar with the guidelines were assigned another group (lack of knowledge, N=114). The results are shown in Table 2.

**Table 2:** **Association of knowledge of German Guidelines and counseling behavior**, **follow up care and knowledge of future health risks**

		**Knowledge of existing guidelines**		**Total**	**Fisher- *t *-test p-Value**
		**No**	**Yes**		
Counseling on self-blood pressure-measurement	No	47 (41.6%)	20 (21.3%)	67 (32.4%)	0.003
	Yes	66 (58.4%)	74 (78.7%)	140 (67.7%)	
	Total	113	94	207	
Routine counseling for cardiovascular risk reduction on patients >50 years	No	40 (36.0%)	18 (19.8%)	58 (28.7%)	0.013
	Yes	71 (64.0%)	73 (80.2%)	144 (71.3%)	
	Total	111	91	202	
Evaluation of family history of preeclampsia	No	42 (37.2%)	15 (16.0%)	57 (27.5%)	0.001
	Yes	71 (62.8%)	79 (84.0%)	150 (72.5%)	
	Total	113	94	207	
Counseling about the meaning and importance of preeclampsia and long term effects	No	27 (23.9%)	8 (8.5%)	35 (16.9%)	0.005
	Yes	86 (76.1%)	86 (91.5%)	172 (83.1%)	
	Total	113	94	207	
Regularly blood pressure-measurement 3 month after delivery	No	40 (35.1%)	10 (10.8%)	50 (24.2%)	<0.01
	Yes	74 (64.9%)	83 (89.2%)	157 (75.8%)	
	Total	114	93	207	
Evaluation of kidney function 3 month after delivery	No	74 (64.9%)	38 (40.9%)	112 (54.1%)	0.001
	Yes	40 (35.1%)	55 (59.1%)	95 (45.9%)	
	Total	114	93	207	
Counseling on the elevated risk of future cardiovascular disease	No	54 (47.8%)	18 (19.1%)	72 (34.8%)	<0.01
	Yes	59 (52.2%)	76 (80.9%)	135 (65.2%)	
	Total	113	94	207	
Knowledge of the elevated risk of hypertension after preeclampsia	No	23 (20.2%)	5 (5.3%)	28 (13.5%)	0.002
	Yes	91 (79.8%)	89 (94.7%)	180 (86.5%)	
	Total	114	94	208	
Knowledge of the elevated risk of stroke after preeclampsia	No	35 (30.7%)	10 (10.6%)	45 (21.6%)	0.001
	Yes	79 (69.3%)	84 (89.4%)	163 (78.4%)	
	Total	114	94	208	
Knowledge of shorter life expectancy	No	32 (28.8%)	17 (18.9%)	49 (24.4%)	0.14
	Yes	79 (71.2%)	73 (81.1%)	152 (75.6%)	
	Total	111	90	201	

There was no significant difference concerning the length of profession (p=0.40) or the mean age of patients that were treated in the outpatient clinic (p=0.096) between the groups.

Physicians that were aware of the guidelines counseled women older 50 years of age more often on the reduction of cardiovascular risk factors than physicians that had no knowledge of the current guidelines (p=0.013).

Obstetricians-gynecologists with knowledge of the guidelines were more often aware of the higher risk for the development of CVD (p=0.002) and stroke (p=0.001) after PE and counseled these patients on self blood pressure measurement (p=0.003), compared to physicians with no knowledge of the guidelines.

Obstetrician-gynecologists having good knowledge about the guidelines attached importance to family history of PE (p=0.001).

With respect to the follow-up care after PE there was a highly significant difference concerning blood pressure measurement three months postpartum (p<0.01). Counseling about meaning and long-term risks of PE (p=0.005) and the development of CVD (p<0.01) was performed more often in the group of physicians with good knowledge of the guidelines, compared to those with lack of knowledge.

There was no significant difference in the knowledge of a shorter life expectancy (p=0.14) between physicians with good knowledge of the guidelines and respondents with lack of knowledge.

## Discussion

In this study we aimed to assess the knowledge of obstetrician-gynecologists in outpatient settings in the public state of Lower Saxony (7.9 Mill. residents in 2012) of the association between PE and future health risk, e.g. the association with renal diseases, stroke and hypertension and the knowledge of the current German guidelines for the treatment and follow-up of women that experienced PE in pregnancy.

Our results suggest that those physicians that answered the survey have a good knowledge about the association between PE and the development of vascular diseases later in life. Although most respondents (86.6%) were aware of the association between PE and future CVD, only 45.2% knew the current guidelines for the long-term treatment and counseling of women with PE. Time of profession and mean age of patients that were treated in the outpatient clinic had no influence on that knowledge. Nearly all respondents included PE as part of their medical history and counseled on the elevated risk for the development of CVD and kidney disease.

Large cohort studies confirm the association of PE with future CVD, stroke and kidney disease later in life although the pathophysiology of PE is still not fully understood.

While the national guideline for the prevention of CVD includes smoking, malnutrition and overweight, hypertension, genetic factors and lipid metabolism disorder as risk factors for the development of future CVD, it does not address the association between PE and future CVD [19].

To our knowledge only a few studies have looked into awareness and knowledge of physicians on long-term health risks after PE, long-term treatment after PE and the knowledge of current national guidelines. Macdonald et al. evaluated the communication between maternity care providers in Ontario and the knowledge about the association between PE and the elevated risk for the development of CVD later in life. In that study only 54.0% of respondents were familiar with the increased risk of CVD after PE [8].

Brett et al. also noted in their study that there was a limited knowledge of the association between PE and future CVD [21]. Most specialists in internal medicine (95.0%) and gynecology (70.0%) provided routine counseling on cardiovascular risk-reduction, but a substantial proportion of them were unaware of any association with a history of PE. About half of the participating internists were not sure or did not know about the association of PE with CVD (56.0%), stroke (48.0%) and decreased life expectancy. Compared to that 23.0% of the gynecologists had lack of knowledge of the risk of the development of CVD, 38.0% were unsure about the stroke risk and 77.0% were unaware of the decreased life expectancy. Of all gynecologists 38.0% were providing cardiovascular risk reduction counseling on women with a history of PE compared to 9.0% of internists in that study.

Our study has a few limitations. Because only some half of the physicians approached, took part in this study, it is unknown whether the knowledge of the non-respondents is comparably good. Furthermore, respondents´ answers may have been influenced by how they suspected the authors wanted them to answer, and if this did occur, the level of knowledge demonstrated here might be artificially high. Due to low numbers in some of the four answer categories we had to group the positive and negative results to be able to perform statistical analysis. This might have caused imprecision and precluded us from a more refined analysis.

Adequate risk reduction counseling can only occur if physicians are aware of the association between PE and long-term health risks and the knowledge of current guidelines. In our study 96.2% of participants indicated an interest in further training. These data suggest appropriate offers for continuing education are necessary and should be established.

## Conclusion

Pregnancy and pregnancy history may be considered as an important screening opportunity for risk factors, identifying women who are at elevated risk of CVD, kidney disease and also diabetes later in life. Women, who experienced PE, might benefit from adequate long-term follow-up care in terms of earlier diagnosis, intervention, life-style changes and risk factor modifications to reduce the long-term morbidity and mortality.

The present study demonstrates that gynecologists and obstetricians in Lower Saxony have a relatively high degree of knowledge with respect to the relationship of PE and long-term health risks, e.g. CVD. The fact that practitioners that knew the recommendations for postpartum care of PE patients, are more often aware of the association between PE and CVD and perform treatment and counseling accordingly, underlines the impact of guidelines on physician's behaviour and treatment strategies.

However, a low level of knowledge of current guidelines of post-PE treatment and counseling of patients was observed, which points to the need for further academic training, as well as continuing education, concerning integrated approaches to health care.

## Competing interests

The authors declare that they have no competing interests.

## Authors’ contributions

MBH, DW and FV-H conception and design of research, DW provided methodological support, MBH, DW and FV-H analyzed data and interpreted results, MBH prepared figures, MBH and FV-H drafted manuscript, FV-H, CS and CSV-K edited and revised manuscript, all authors approved the final version of the manuscript.

## Pre-publication history

The pre-publication history for this paper can be accessed here:

http://www.biomedcentral.com/1471-2393/13/61/prepub

## Supplementary Material

Additional file 1 Supplement Questionnaire. Click here for file
