# Supplementary material for: Preeclampsia and long-term risk of cardiovascular disease: what do obstetrician-gynecologists know?
Source: BMC Pregnancy Childbirth. 2013 Mar 9;13:61. doi: 10.1186/1471-2393-13-61 (PMC3605330; doi:10.1186/1471-2393-13-61)
Supplement: Additional file 1 — Supplement Questionnaire. [file 1471-2393-13-61-S1.pdf]

>

Preeclampsia

- Follow-up treatment and long term risks -

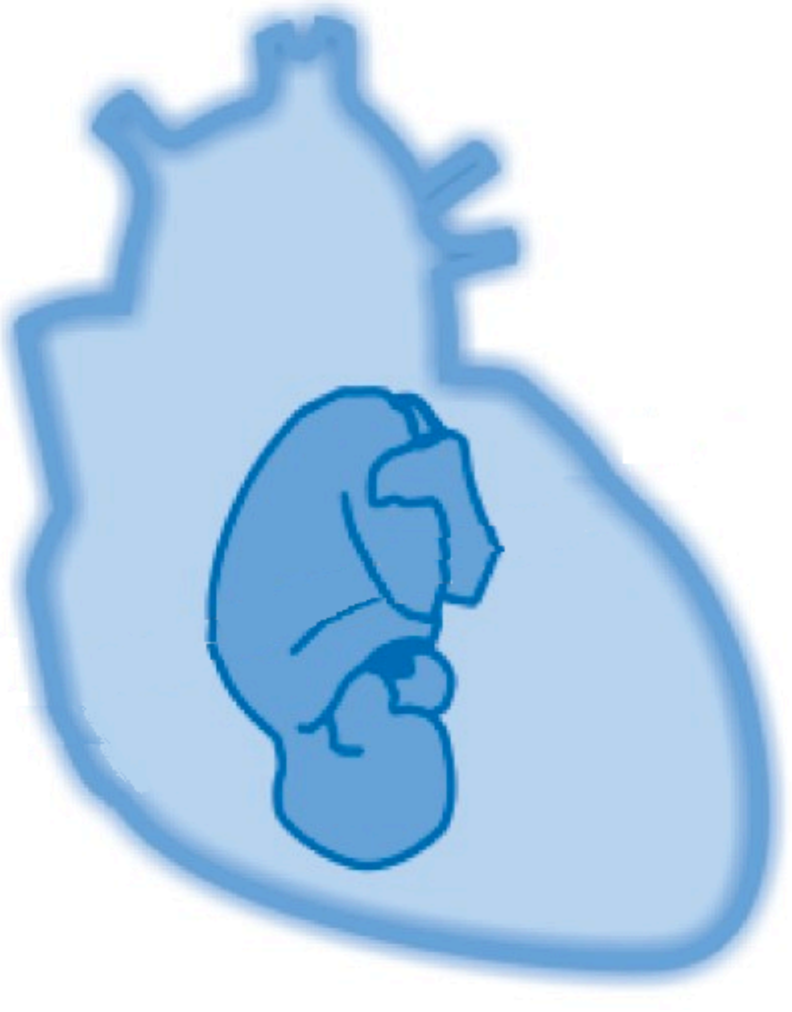

Survey

1. Years of your clinical experience?

   ☐ < 5 years ☐ 6-11 years ☐ 11-20 years ☐ >21 years

2. Your sex

   ☐ male ☐ female

3. Average age of patients you treat

   ☐ 20-40 years ☐ 40-50 years ☐ >50 years

4. Proportion of treated patients > 50 years

   ☐ < 10 % ☐ 11-30% ☐ 31-50% ☐ > 51%

	Definitely do	May do	Hardly do	Definitely don`t do
5. When you perform routine counseling for cardiovascular risk reduction, you				
5.1. give instructions for lifestyle change (weight-reduction)	◯	◯	◯	◯
5.2. counsel on self-help-groups	◯	◯	◯	◯
5.3. counsel on routinely blood-pressure measurement	◯	◯	◯	◯
6. You perform routine counseling for cardiovascular risk reduction on patients >50 years	◯	◯	◯	◯

7. Your patient-history contains	Always	Often	Rarely	Never
7.1. a history of preeclampsia	◯	◯	◯	◯
7.2. a family history of preeclampsia	◯	◯	◯	◯
7.3. hypertension before pregnancy	◯	◯	◯	◯
8. Do you ask for a history of preeclampsia on non-pregnant women?	◯	◯	◯	◯
9. Do you inform your patients about the meaning and importance of preeclampsia and the possible long-term effects?	◯	◯	◯	◯
10. Do you ask patients with a history of preeclampsia for the time of occurrence of preeclampsia?	◯	◯	◯	◯

11. Do you typically counsel on the following points during your follow -up management of patients with a history of preeclampsia?	Always	Often	Rarely	Never
11.1. regularly blood pressure-measurement 3 month after delivery	◯	◯	◯	◯
11.2. evaluation of kidney-disease 3 month after delivery	◯	◯	◯	◯
11.3. ascertainment of serum creatinine and proteinuria	◯	◯	◯	◯
11.4. counseling on the elevated risk of the development of cardiosvascular diseases later in life	◯	◯	◯	◯
11.5. information about the regulary follow-up treatment of the newborn child	◯	◯	◯	◯
12. Do you counsel about the elevated risk of the development of cardiosvascular disease later in life?	◯	◯	◯	◯

13. How familiar are you with the elevated risk of women with a history of preeclampsia to develop the following diseases?	Very well	Well	Less well	Not at all
13.1. malignancies	◯	◯	◯	◯
13.2. hypertension	◯	◯	◯	◯
13.3. stroke	◯	◯	◯	◯
13.4. kidney disease	◯	◯	◯	◯
13.5. liver disease	◯	◯	◯	◯
14. How familiar are you with the current guidelines of the DGGG concerning treatment and follow-up management of preeclampsia?	◯	◯	◯	◯

	Definitely applies	May apply	Propably doesn´t apply	Definitlely doesn`t apply
15. Do you think women with a history of preeclampsia have a shorter life expectancy?	◯	◯	◯	◯

	Very much	Much	Not much	Not at all
16. How much are you interested in further education on the topic preeclampsia?	◯	◯	◯	◯

Thank you for your cooperation
